# Deep inferior epigastric perforator flap demonstrating the triple response of Lewis phenomenon – A case report and insights into aetiology

**DOI:** 10.1016/j.jpra.2019.11.008

**Published:** 2019-12-26

**Authors:** Mohammad Malik, Zita M Jessop, Mark Cooper

**Affiliations:** a Burns and Plastics Department, Morriston Hospital, Heol Maes Eglwys, Morriston, Cwmrhydyceirw, Swansea SA6 6NL, United Kingdom; b Reconstructive Surgery and Regenerative Medicine Research Group Swansea University Medical School, Swansea SA2 28PP, United Kingdom

**Keywords:** Dermatographia, DIEP, Free Flap

## Abstract

Dermatographism, also known as skin writing, is an immunological response that remains an enigma. It occurs when normal skin is stroked with a dull object causing it to become raised and inflamed, and assume the shape of the stroke. Here we present a case of dermatographia in a denervated deep inferior epigastric perforator flap used to reconstruct breast tissue following mastectomy.

## Introduction

Dermatographism, also known as skin writing, is an immunological response that remains an enigma. It occurs when normal skin is stroked with a dull object, causing it to become raised and inflamed and assume the shape of the stroke. It is a yet incompletely understood phenomenon with an undetermined clinical significance.

The diagnosis of dermatographism is clinical. Application of moderate pressure or a scratch elicits the reaction.^1^ The prevalence is believed to be between 1.5 and 5% in the general population.^2^, ^3^, ^4^, ^5^, ^6^ The phenomenon is more pronounced in areas that are regularly protected from pressure or environmental exposure. The trunk is therefore more often implicated than the limbs. Previously, there has been no recorded cases of dermatographia in a free flap. Here we present a case of dermatographia in a free flap following a deep inferior epigastric perforator (DIEP) flap reconstruction.

## Case report

Mrs C is a 50 year old female who underwent a left sided mastectomy in 2014 for breast cancer, followed by radiotherapy that completed in February 2015. Her only other past medical history of note is diabetes, for which she takes metformin and gliclazide. Following discussion of the reconstructive options she opted for a delayed left DIEP flap reconstruction.

**Figure 1:**
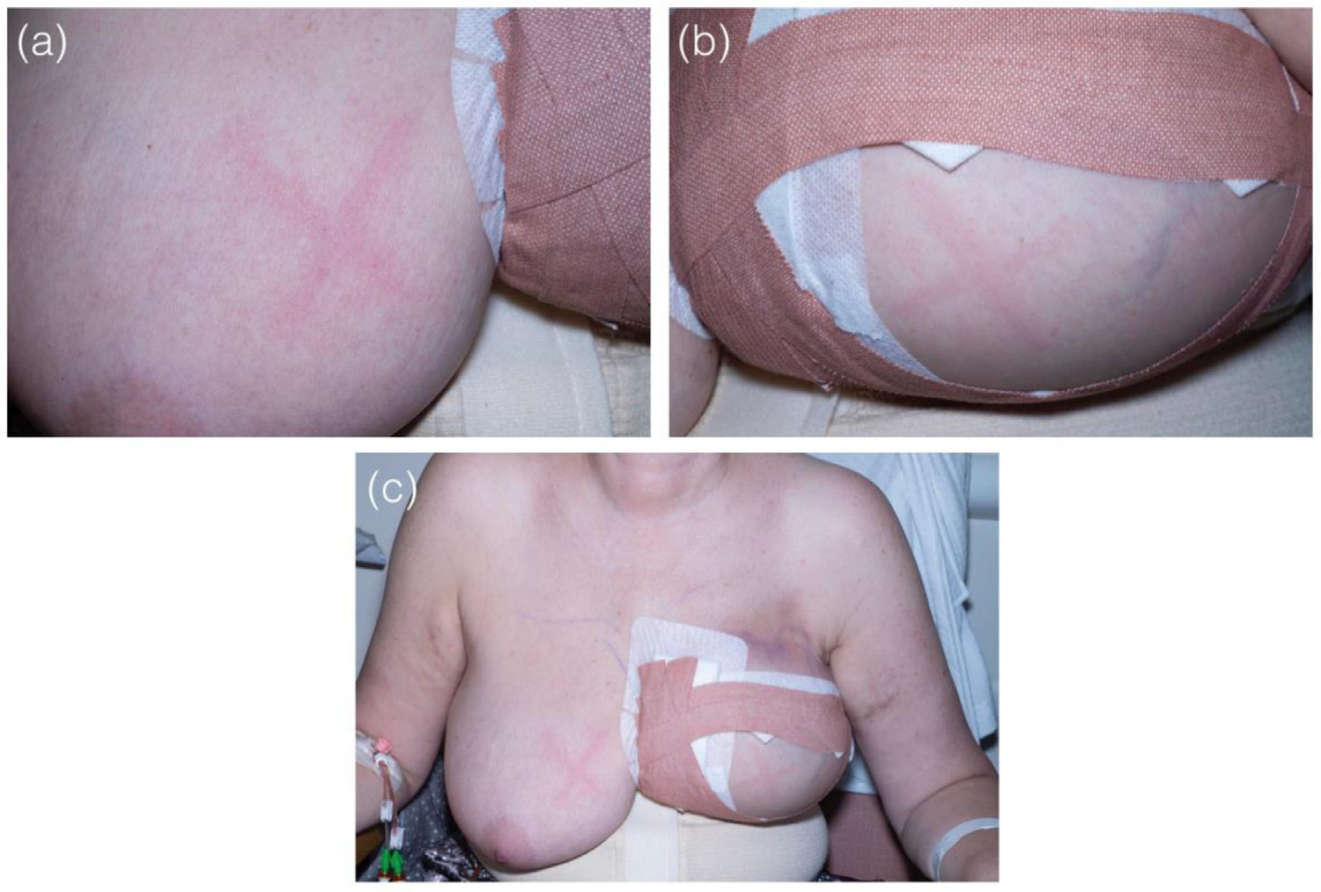
Photographs demonstrating characteristic dermatographia. (a) Right breast, (not operated) on. (b) Left breast (operated on). (c) Comparison of the response in the two breasts.

She underwent an uncomplicated delayed DIEP reconstruction and was subsequently transferred to the flap monitoring unit for ongoing assessment of the flap. During her inpatient stay, her DIEP flap was lightly scratched using the tip of a nail as part of flap monitoring to assess capillary refill time. The flap immediately demonstrated the classic red linear indurated streaks characteristic of dermatographia, this was then compared to a scratch on the other breast (see Figure 1).

Following an uncomplicated post-operative period, she was subsequently discharged to routine follow up. At 6 month follow up, the test was repeated, using light stroke on both the DIEP flap and non-operated side, the triple response of Lewis was still evident.

## Discussion

Although incompletely understood, Dermatographism is thought to be an exaggerated triple response of Lewis. Originally described in 1928,^7^ scratching of the skin produces an initial red line due to capillary dilatation. This is then followed by an axon reflex flare that causes broadening erythema secondary to arteriolar dilatation and finally a linear wheal by the transudation of fluid and oedema.^8^^,^^9^ A normal triple response of Lewis occurs slowly and subsides within 5–10 min, whereas dermatographism occurs almost immediately and can last for 15–30 min.^10^ Underlying mechanisms are thought to be mechano-immunological, whereby mechanical stress stimulates the degranulation of mast cells, causing the release of histamine, as well as other inflammatory mediators, stimulating blood vessel permeability and fluid migration into the tissues.^8^, ^9^, ^10^

**Figure 2:**
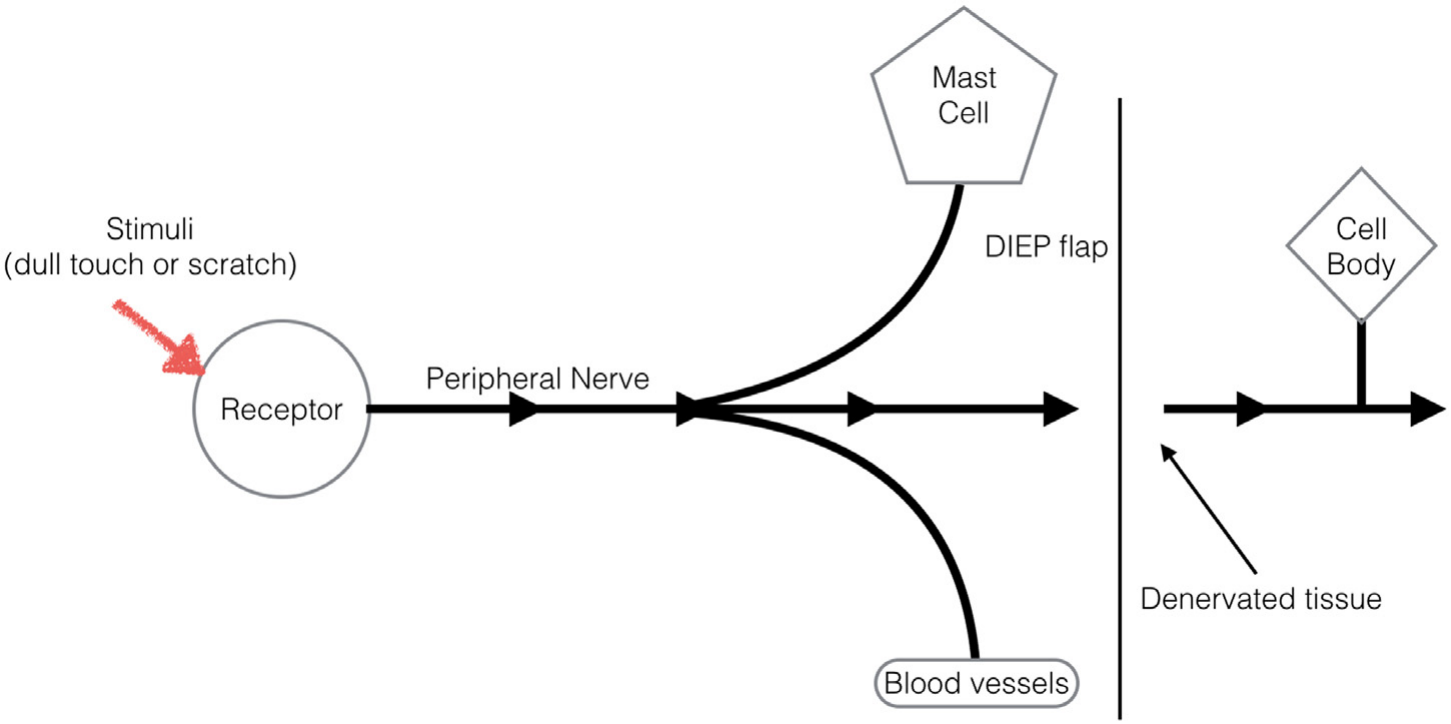
Diagram demonstrating the peripheral nerve pathway of a sensory nerve in a dennervated free flap. Although no longer in continuity, there is signal transmission to effect organs prior to synapse, allowing for the Triple Response of Lewis.

This case demonstrates that even without peripheral nerve stimulation, the response can occur. Previous evidence in the literature demonstrates that dennervated tissue has the potential to demonstrate the triple response of Lewis.^8^This is because the axon reflex transmits signals directly to the effector organ, bypassing both an integration centre and chemical synapse, allowing it to stimulate cells around the nerve itself, including other nerve axons, gland, muscle tissue and arterioles (see Figure 2). Hence the presence of dermatographism in a dennervated free flap.

## Conclusion

Although the low incidence of dermatographia^2^, ^3^, ^4^, ^5^, ^6^ in the general population and variation in response between patients who do demonstrate this phenomenon will mean that this is not necessarily a useful way of monitoring free flaps, it does provide some interesting insights into the aetiology of the condition.

## Declaration of Competing Interest

None.
